# Total tau in cerebrospinal fluid detects treatment responders among spinal muscular atrophy types 1–3 patients treated with nusinersen

**DOI:** 10.1111/cns.14051

**Published:** 2022-12-13

**Authors:** Goran Šimić, Vana Vukić, Marija Babić, Maria Banović, Ivana Berečić, Ena Španić, Klara Zubčić, Anja Tea Golubić, Marija Barišić Kutija, Ana Merkler Šorgić, Željka Vogrinc, Ivan Lehman, Patrick R. Hof, Jadranka Sertić, Nina Barišić

**Affiliations:** ^1^ Department of Neuroscience, Croatian Institute for Brain Research University of Zagreb School of Medicine Zagreb Croatia; ^2^ Department of Pediatrics University Hospital Centre Zagreb Zagreb Croatia; ^3^ Department of Nuclear Medicine and Radiation Protection University Hospital Centre Zagreb Zagreb Croatia; ^4^ Department of Ophthalmology University Hospital Centre Zagreb Zagreb Croatia; ^5^ Department of Laboratory Diagnostics, Laboratory for Molecular Diagnostics University Hospital Centre Zagreb Zagreb Croatia; ^6^ Department of Laboratory Diagnostics University Hospital Centre Zagreb Zagreb Croatia; ^7^ Nash Family Department of Neuroscience, Friedman Brain Institute, and Ronald M. Loeb Center for Alzheimer's Disease Icahn School of Medicine at Mount Sinai New York New York USA; ^8^ Department of Medical Chemistry and Biochemistry University of Zagreb School of Medicine Zagreb Croatia

**Keywords:** biomarker, nusinersen, spinal muscular atrophy, tau protein

## Abstract

**Aims:**

Considering the substantial variability in treatment response across patients with spinal muscular atrophy (SMA), reliable markers for monitoring response to therapy and predicting treatment responders need to be identified. The study aimed to determine if measured concentrations of disease biomarkers (total tau protein, neurofilament light chain, and S100B protein) correlate with the duration of nusinersen treatment and with scores obtained using functional scales for the assessment of motor abilities.

**Methods:**

A total of 30 subjects with SMA treated with nusinersen between 2017 and 2021 at the Department of Pediatrics, University Hospital Centre Zagreb, Croatia, were included in this study. Cerebrospinal fluid (CSF) samples were collected by lumbar puncture prior to intrathecal application of nusinersen. Protein concentrations in CSF samples were determined by enzyme‐linked immunosorbent assay in 26 subjects. The motor functions were assessed using functional motor scales.

**Results:**

The main finding was significantly decreased total tau correlating with the number of nusinersen doses and motor improvement in the first 18–24 months of treatment (in all SMA patients and SMA type 1 patients). Neurofilament light chain and S100B were not significantly changed after administration of nusinersen.

**Conclusions:**

The measurement of total tau concentration in CSF is a reliable index for monitoring the biomarker and clinical response to nusinersen therapy in patients with SMA.

## Graphical abstract

In this study, we performed a follow‐up study of 26 spinal muscular atrophy types 1–3 patients treated with nusinersen between 2017 and 2021 to determine if measured concentrations of total tau, neurofilament light chain, and S100B proteins in cerebrospinal fluid correlate with the duration of nusinersen treatment and with scores obtained using functional scales of motor abilities. The main finding was significantly decreased total tau protein correlating with the number of nusinersen doses and motor improvement in the first 18–24 months of treatment. Neurofilament light chain and S100B were not significantly changed after administration of nusinersen.

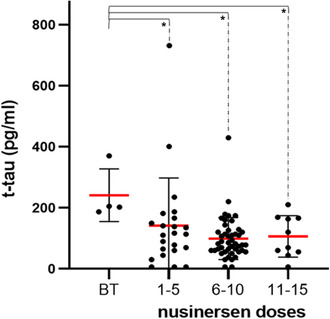

## 1 INTRODUCTION

Proximal spinal muscular atrophy (SMA) is a progressive autosomal recessive neurodegenerative disease with an incidence of 1 in 6–11,000 live births.^1^ It is caused by homozygous deletion or mutation of the *SMN1* gene (survival motor neuron 1, telomeric) on chromosome 5q11.2–q13.3. The leading sign is progressive muscle weakness and atrophy. Depending on the age of onset of the first signs, the disease is clinically classified into five phenotypes; the most common type of SMA that occurs in the first 6 months, SMA type 1 (Werdnig‐Hoffmann disease), SMA type 2 (the first signs manifest at the age of 7–18 months), SMA type 3 (Kugelberg‐Welander disease) that occurs after 18 months and is subdivided into 3a that manifests before 3 and 3b that manifests after 3 years of age, SMA type 4 with the onset in the second or the third decade of life, and SMA type 0 that manifests with fetal hypomotility or akinesia prenatally.^2^

Patients with SMA have a mutation or deletion of the *SMN1* gene, with all patients having at least one copy of the *SMN2* (survival motor neuron 2, centromeric) gene as they would not survive in utero without it. It seems that the *SMN2* gene is unique to humans.^3^ Pathological changes in SMA consist of the loss of anterior horn neurons in the spinal cord (primarily α‐motoneurons, as well as interneurons and γ‐motoneurons) and sometimes loss of bulbar motoneurons, empty cell beds, bundles of glial cells in the ventral roots of the spinal cord, and heterotopic, migratory motoneurons.^3^, ^4^, ^5^

The *SMN* gene on chromosome 5 is a duplicate gene that has two highly homologous copies, a telomeric copy called *SMN1* and a centromeric copy known as the *SMN2* gene. *SMN2* differs from *SMN1* in cytosine substitution by thymine in exon 7 (840C<T), which is sufficient to silence the inclusion of exon 7 in the *SMN2* gene.^6^ The *SMN1* gene provides a full‐length functional SMN protein, and in healthy individuals, its activity is dominant and sufficient. Homozygous disruption of the *SMN1* gene due to deletion of exon 7 or gene conversion of *SMN1* to *SMN2* leads to the development of SMA in about 95% of cases.^7^ About 3% of SMA patients are compound heterozygotes for deletion of one *SMN1* allele and small intragenic mutations. However, the type of SMA, i.e., the time of onset and severity of clinical signs, also depends on the involvement of surrounding genes in the 5q13 region as well as some other genes located on other chromosomes. Among the listed disease‐modifying genes, *SMN2* stands out as the most important. Although it is responsible for the production of only 10% of functional SMN protein in healthy people, in case of a deletion of the *SMN1* gene, its activity is essential for survival.^8^, ^9^ Unlike *SMN1*, the number of copies of the *SMN2* gene is variable and there may be more than one copy of the gene. The greater the number of *SMN2* copies, typically milder the SMA phenotype that is also less progressive and has a later onset. About 80% of patients with SMA type 1 have one or two *SMN2* copies. About 82% of patients with SMA type 2 carry three *SMN2* copies, while 96% of patients with SMA type 3 have three or four copies of this gene.^10^ The fact that other genes also influence SMA severity^11^ may explain why other studies did not observe a correlation between SMN levels and SMA severity.^12^ *SMN2* gene variant c.859G>C in exon 7 causes a milder phenotype as it increases the inclusion of exon 7. In addition to *SMN2*, other disease‐modifying genes have been found as well, one of which is the NLR family apoptosis inhibitory protein (*NAIP*)/*BIRC1* gene, encoding a protein from the NLR family of apoptosis inhibitors. Although the role of the *NAIP* gene in SMA has not yet been fully elucidated, it is associated with the severity of the clinical presentation. Patients with a homozygous SMN1 exon 7 deletion who do not have a copy of the *NAIP* gene have a more severe phenotype: in 75% of patients with SMA type 1, the *NAIP* gene is absent.^13^ Additional SMA‐modifying genes are *PLS3* (plastin 3),^14^ *CORO1C* (coronin 1C),^15^ and others.^16^

The discovery of the intronic splice silencing site (ISS‐N1) in 2004 led to the first SMA disease‐modifying treatment, nusinersen, and antisense oligonucleotide [2'‐O‐(2‐methoxy)‐ethyl‐phosphorothioate] for intrathecal administration, approved by the U.S. Food and Drug Administration (FDA) in 2016.^17^ Later, the FDA approved gene replacement therapy onasemnogene abeparvovec for the treatment of SMA in children under 2 years of age, designed as one dose for life, administered intravenously.^18^, ^19^ Also, a small‐molecule risdiplam, a pyridazine derivative administered orally, acts similar to nusinersen by increasing the inclusion of exon 7 in the mRNA of the patient's *SMN2* gene.^16^

Nusinersen targets the ISS‐N1 sequence in intron 7 of the *SMN2* gene that prevents the inclusion of exon 7 in *SMN2* mRNA. When nusinersen binds to the intronic splicing silencer (ISS)‐N1 sequence, it allows the inclusion of exon 7 in *SMN2* mRNA, and consequently, increases the production of full‐length SMN protein.^16^, ^17^

SMN protein is involved in the biogenesis of small nuclear ribonucleoproteins and mRNA splicing and probably has a major role in motoneuron cellular as well as neuromuscular junction functions. SMN protein levels are significantly reduced in cells of the central and peripheral nervous system in SMA patients.^19^, ^20^, ^21^ SMN protein levels decrease with age in humans and most other species, emphasizing the importance of early treatment interventions. Current disease‐modifying treatments increase SMN protein levels by targeting *SMN2* pre‐mRNA splicing (nusinersen and risdiplam) or by replacement of fully functional recombinant *SMN1* transgene (onasemnogene abeparvovec).

Neurofilaments (Nf), especially phosphorylated Nf heavy‐chain (pNFH) and light‐chain Nf (NfL) levels have emerged as potential biological markers in monitoring SMA progression and response to nusinersen therapy in infants.^21^ Elevated levels of NfL in the CSF reflect neuronal and axonal degeneration^22^; therefore, it is not a disease‐specific biomarker.^23^ In addition to Nf, serum creatinine and creatine kinase may also correlate well with motor function and disease severity, and their measurement in the blood might be useful to determine patients who will respond to therapy.^24^

Other, potentially useful biological markers for monitoring response to nusinersen are total tau (t‐tau) protein and S100 calcium‐binding protein B (S100B).^25^ Tau proteins maintain the stability of microtubules. The increase in the concentration of t‐tau protein in the CSF occurs due to neuronal death and axonal degeneration.^26^ An increase in the concentration of t‐tau protein has been observed in several neurodegenerative diseases, especially Alzheimer's disease^4^, ^26^, ^27^, ^28^, ^29^, ^30^, ^31^, ^32^, ^33^ and Creutzfeldt–Jakob disease.^34^

S100 proteins belong to the large family of small dimeric multigenic calcium‐binding proteins.^35^ S100B is specific for astrocytes, especially mature astrocytes whose end‐feet are crucially involved in the formation of the blood–brain barrier (glia limitans).^36^ An increase in S100B occurs in many diseases of the central nervous system such as Alzheimer's disease, Parkinson's disease, amyotrophic lateral sclerosis, and multiple sclerosis, while elevated S100B levels in the peripheral nervous system indicate inflammatory autoimmune diseases or axonal degeneration and regeneration.^37^

While some studies showed that NfL levels decrease in SMA patients upon nusinersen treatment,^38^, ^39^, ^40^, ^41^, ^42^ data on S100B and t‐tau are quite variable. Only one study measured S100B levels in patients with SMA type 3, showing no difference in S100B after nusinersen treatment.^25^ Two studies, including mainly SMA type 1 patients, observed a significant decrease in t‐tau levels upon nusinersen treatment, whereas others, including mainly SMA type 2 and type 3 patients, did not report changes in t‐tau levels after nusinersen.^25^, ^43^, ^44^ Thus, we aimed to test the potential of t‐tau, NfL, and S100B proteins as theragnostic biomarkers in SMA. We hypothesized that nusinersen therapy would lead to a decrease in the concentrations of t‐tau, NfL, and S100B proteins in the CSF of SMA patients.

## 2 METHODS

### 2.1 Subjects, genetic analyses, administration of nusinersen, and CSF sampling

All procedures were performed with the approval of the Ethics Committee of the Clinical Hospital Center Zagreb (class: 8.1‐20/185‐2, no. 02/21AG) and by the standards of the Helsinki Declaration and International Standards on Good Clinical Practice (International Conference on Harmonization, 2013).^45^ This longitudinal study included 30 SMA subjects who were treated at the Department of Pediatrics (Clinic of Pediatrics), Division of Neurology at the University Hospital Centre Zagreb (Table 1; 16 SMA type 1, 4 SMA type 2, and 10 SMA type 3 patients), from 2017 to 2021.

**TABLE 1:** Demographics, genetics, and treatment characteristics of SMA subjects

Subject	SMA type	Sex	Age at first dose	Number of nusinersen doses received	Copy number of *SMN1* exon 8 gene	Copy number of *SMN2* exon 7 gene	Copy number of *SMN2* exon 8 gene	Copy number of *NAIP* exon 5 gene	CSF sample	CHOP INTEND or HFMSE
SMA003	1	F	6 months	15	0	2	2	0	+	+
SMA005	1	M	10 years	7	0	2	2	0	+	HFMS
SMA007	1	M	7 months	7	0	2	2	n.a.	+	HFMS
SMA008	1	M	4 months	7	0	2	2	0	+	+
SMA012	1	M	7 months	11	0	2	2	0	+	+
SMA013	1	F	3 years	13	0	3	3	2	+	+
SMA014	1	M	3 months	10	0	2	2	1	+	+
SMA015	1	M	4 months	10	0	2	2	1	+	+
SMA016	1	M	8 years	7	0	2	2	0	+	+
SMA018	1	F	3 years	14	0	2	2	0	+	+
SMA022	1	M	5 years	9	0	2	2	0	+	+
SMA026	1	M	8 years	6	0	3	3	1	+	+
SMA033	1	M	1 months	6	1	2	1	1	+	HFMS
SMA035	1	F	9 years	7	0	2	2	0	n.a.	+
SMA021	1	F	3 years	10	1	2	2	1	n.a.	+
SMA037	1	M	6 years	5	0	2	2	0	n.a.	+
SMA002	2	M	8 years	12	0	3	3	1	+	+
SMA024	2	M	3 years	5	0	3	3	n.a.	+	HFMS
SMA029	2	F	12 years	9	1	1	1	1	+	+
SMA030	2	M	1 years	7	1	3	2	1	+	+
SMA001	3	F	17 years	8	0	4	4	2	+	+
SMA004	3	F	13 years	12	1	3	3	2	+	+
SMA006	3	M	14 years	13	1	3	3	2	+	+
SMA009	3a	M	10 years	12	1	2	2	1	+	+
SMA010	3	M	15 years	12	1	4	3	2	+	+
SMA017	3a	F	5 years	11	0	3	3	2	+	+
SMA019	3b	F	8 years	12	0	3	4	2	+	+
SMA028	3a	F	4 years	11	0	2	3	0	+	+
SMA031	3b	M	12 years	10	0	4	4	2	+	+
SMA036	3b	M	17 years	7	0	3	3	2	n.a.	+

Abbreviations: CHOP INTEND, Children's Hospital of Philadelphia Infant Test of Neuromuscular Disorders; F, Female; HFMS, Hammersmith Functional Motor Scale; HFMSE, Hammersmith Functional Motor Scale Expanded; M, Male; m, months; n.a., not available; NAIP, NLR Family Apoptosis Inhibitory Protein; SMA, spinal muscular atrophy; SMN, survival motor neuron; y, years.

Genetic analyses were performed at the Department of Molecular Laboratory Diagnostics of the Clinical Institute for Laboratory Diagnostics, Clinical Hospital Center Zagreb, by multiplex ligation‐dependent probe amplification (MLPA). SALSA MLPA kit MRC – P021‐B1 SMA, Applied Biosystems capillary sequencer, and the publicly available Coffalyser.Net (MRC‐Holland, Amsterdam, The Netherlands) MLPA analysis software were used.^46^, ^47^, ^48^, ^49^ Genomic DNA was isolated from a peripheral whole blood sample. The laboratory participates in the quality control scheme EMQN.

CSF samples were recovered from 13 SMA type 1, 4 SMA type 2, and 9 SMA type 3 patients. Each subject underwent a thorough neurological examination. Healthy subjects were not included in the study. CSF samples were taken upon the informed consent of a parent or legal guardian. CSF samples (5 ml) were collected by spinal tap (lumbar puncture) from the L3/L4 intervertebral space just before nusinersen administration. A small portion of CSF (1 ml) was aliquoted and stored at −80°C in polypropylene tubes for later analysis (Table 2). However, we were not able to collect CSF for analysis of biomarkers after the administration of every nusinersen dose. The time points at which CSF was collected for analysis of biomarkers are indicated in Table 2.

**TABLE 2:** Time points of CSF samples collection (indicated by +) and the total number of doses received

Subject	Nusinersen dose number															Total number of nusinersen doses received
	0	1	2	3	4	5	6	7	8	9	10	11	12	13	14	
SMA001	+			+												8
SMA002							+	+	+							12
SMA003										+		+	+	+	+	15
SMA004							+	+			+					12
SMA005					+	+	+									7
SMA006								+			+		+			13
SMA007	+					+	+									7
SMA008					+	+	+									7
SMA009							+	+			+	+				12
SMA010								+	+							12
SMA012						+	+			+						11
SMA013									+	+	+					13
SMA014								+	+	+						10
SMA015					+		+		+							10
SMA016					+	+	+									7
SMA017							+		+	+	+	+				11
SMA018									+		+	+	+			14
SMA019							+		+	+	+	+				12
SMA022					+		+	+								9
SMA024			+	+												5
SMA026			+	+	+											6
SMA028								+	+	+						11
SMA029			+		+	+										9
SMA030	+				+		+									7
SMA031						+		+		+						10
SMA033	+			+	+											6

*Note*: CSF for analysis of biomarkers was not collected during the administration of every nusinersen dose.

### 2.2 Assessment of motor functions

The motor abilities of SMA patients were measured using the Children's Hospital of Philadelphia Infant Test of Neuromuscular Disorders (CHOP INTEND) functional scale (score 0–64)^50^ and the Hammersmith Functional Motor Scale Expanded (HFMSE) scale (score 0–66).^51^ Patients with SMA type 1 and type 2 were tested using both the CHOP INTEND and HFMSE, as recommended for children up to the age of 2 years and older/>2 years, non‐ambulant were assessed by CHOP INTEND, whereas children who scored 50 or more CHOP INTEND points were additionally tested using HFMSE. SMA patients older than 2 years of age with SMA types 2 and 3 were tested using the HFMSE only (a small number of the patients were sometimes also tested using the HFMS (Hammersmith Functional Motor Scale)).^52^ It is recommended that all patients with SMA be neurologically examined at baseline prior to the first initial four doses and every 4 months afterward before each successive dose (5th, 6th, etc.), which means four times in the first year and three times per each successive year, and the examination should include an assessment of motor functions measurements based on functional scales as well as breathing functions.^53^

### 2.3 Measurement of t‐tau, NfL, and S100B concentrations in CSF by using ELISA

Concentrations of specific proteins in CSF were determined by enzyme‐linked immunosorbent assay (ELISA). Commercially available kits for t‐tau (Innotest hTau Ag, Fujirebio, Tokyo, Japan), NfL (Human Nf‐L/NEfL ELISA Kit, LifeSpan BioSciences Inc.), and S100B (Human S100B, R&D Systems) were used.

### 2.4 Statistical analysis

Statistical analysis was performed with SPSS, version 19.0.1 (SPSS). The statistical significance level was set at α = 0.05 for all tests. The normality of the distribution of individual variables within the groups was tested using the Kolmogorov–Smirnov test. Additionally, due to the small number of subjects, non‐parametric Kruskal–Wallis and Mann–Whitney tests were used. The relationship between the tested variables (dependent variables) and the received number of nusinersen doses (independent variable) was tested using linear regression. Outliers were not included in the statistical analysis. A comparison of genetic data among patients with different types of SMA was performed using the *χ* ^2^ test. Given the exploratory nature and design of the study, no correction for multiple testing was performed.

## 3 RESULTS

### 3.1 Comparison of t‐tau protein levels in patients with SMA of all types

**FIGURE 1:**
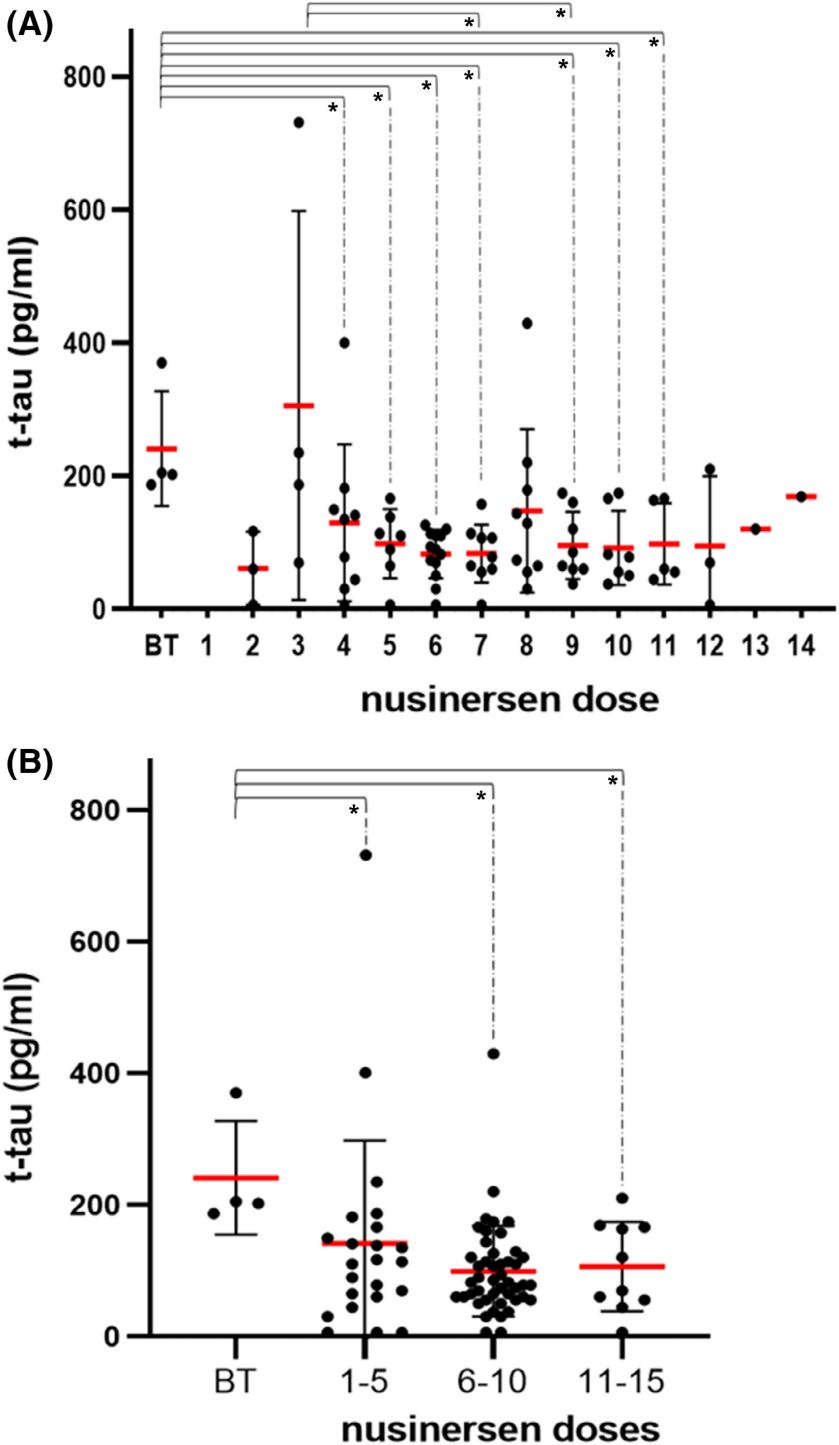
(A) Concentrations of t‐tau protein in patients with SMA 1, SMA 2, and SMA 3 who received different doses of nusinersen. B. Levels of t‐tau protein after grouping the patients with SMA 1, SMA 2, and SMA 3 according to the number of doses received. Each patient is represented by a dot on the graph. The red horizontal line represents the mean value, while the black horizontal lines represent the standard deviation. *p < 0.05

Linear regression showed a statistically significant negative association between t‐tau protein concentrations (dependent variable) and nusinersen dose values (independent variable, β = −0.23; SE = 3.64; *p* = 0.04, Figure 1A,B). T‐tau protein levels were significantly reduced in all SMA patients (SMA 1, SMA 2, and SMA 3) who received the 4th dose of nusinersen compared to baseline values. The concentrations of t‐tau protein were significantly reduced in patients who received the 5th, 6th, 7th, 9th, 10th, and 11th dose of the drug compared to baseline values (Figure 1A). T‐tau protein levels were significantly reduced in patients who received the 7th and 9th dose of the drug compared to those who received the 3rd dose of the drug (Figure 1A).

As means of grouped data are more accurate than means of ungrouped data, we analyzed patient data in four groups: before treatment (baseline values), 1–5 doses, 6–10 doses, and 11–15 nusinersen doses (Figure 1B). There was an overall significant difference in the concentrations of t‐tau protein (*H* test = 9 0.55; *df* = 3; *p* = 0.02). T‐tau protein levels were significantly lower in the group of all SMA patients (SMA 1, SMA 2, and SMA 3) who received 1–5 doses compared to baseline values, as well as in patients who received 6–10 doses and 11–15 doses compared to baseline values (Figure 1B; Table 3).

**TABLE 3:** The results of statistical comparison of t‐tau levels between analyzed groups

Tested groups	The result of the statistical test
SMA 1, SMA 2, and SMA 3	
BT vs. 4th dose	*U* = 4; *Z* = −2.16; *p* = 0.03
BT vs. 5th dose	*U* = 0; *Z* = −2.65; *p* = 0.01
BT vs. 6th dose	*U* = 0; *Z* = −2.95; *p* < 0.01
BT vs. 7th dose	*U* = 0; *Z* = −2.78; *p* < 0.01
BT vs. 9th dose	*U* = 0; *Z* = −2.72; *p* < 0.01
BT vs. 10th dose	*U* = 0; *Z* = −2.65; *p* = 0.01
BT vs. 11th dose	*U* = 0; *Z* = −2.45; *p* = 0.02
3rd dose vs. 7th dose	*U* = 5; *Z* = −2.01; *p* = 0.05
3rd dose vs. 9th dose	*U* = 4; *Z* = −2.04; *p* = 0.04
BT vs. 1st–5th dose	*U* = 11.50; *Z* = −2.36; *p* = 0.01
BT vs. 6th–10th dose	*U* = 7; *Z* = −3.04; *p* < 0.01
BT vs. 11th–15th dose	*U* = 3; *Z* = −2.04; *p* = 0.01
BT vs. 0–6 months	*U* = 11.5; *Z* = −2.729; *p* = 0.003
BT vs. 6–12 months	*U* = 7; *Z* = −2.471; *p* = 0.01
BT vs. 12–18 months	*U* = 0; *Z* = −3.004; *p* = 0.001
BT vs. 18–24 months	*U* = 0; *Z* = −2.449; *p* = 0.016
SMA 1	
BT vs. 4th–6th dose	*U* = 2; *Z* = −2.04; *p* = 0.03
BT vs. 7th–9th dose	*U* = 1; *Z* = −1.94; *p* = 0.05
BT vs. 6th–10th dose	*U* = 1; *Z* = −2.16; *p* = 0.02
BT vs. 12–18 months	*U* = 0; *Z* = −2.012; *p* = 0.044

Abbreviations: BT, before therapy; SMA, spinal muscular atrophy.

### 3.2 Comparison of t‐tau protein levels in SMA patients in relation to the duration of treatment and SMA type

**FIGURE 2:**
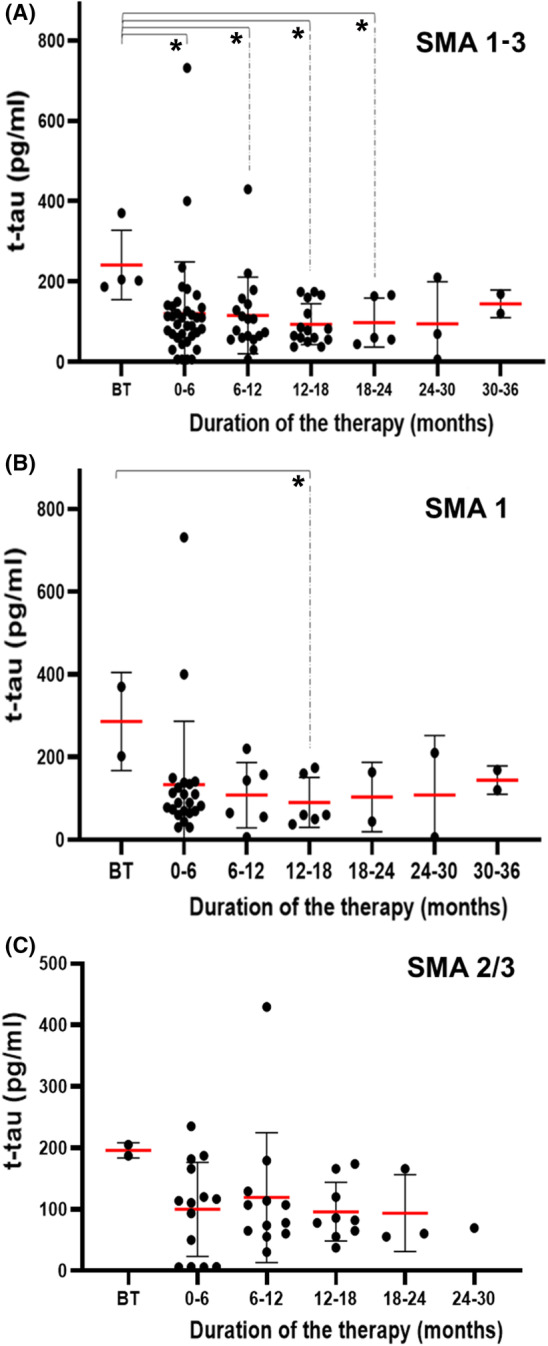
T‐tau protein concentrations in (A) all SMA patients, (B) SMA 1 patients, and (C) SMA 2/3 patients grouped based on nusinersen treatment duration. Each patient is represented by a dot on the graph. The red horizontal line represents the mean value, while the black horizontal lines represent the standard deviation. *p < 0.05

Similar to pooling the number of doses, we grouped the patients who were receiving nusinersen into six groups: 0–6 months, 6–12 months, 12–18 months, 18–24 months, 24–30 months, and 30–36 months. The rationale for such division into subgroups was the assumption that half‐year periods were sufficiently long to detect significant changes if they occur. In comparison to baseline values, the results for all 30 SMA patients taken together were highly significant for all subgroups up to the 18–24 months group (Figure 2A; Table 3), whereas the results for SMA type 1 subjects only, probably due to a lower number of cases analyzed, were significant for the 18–24 months group only (Figure 2B).

### 3.3 Comparison of NfL and S100B protein levels in SMA patients

**FIGURE 3:**
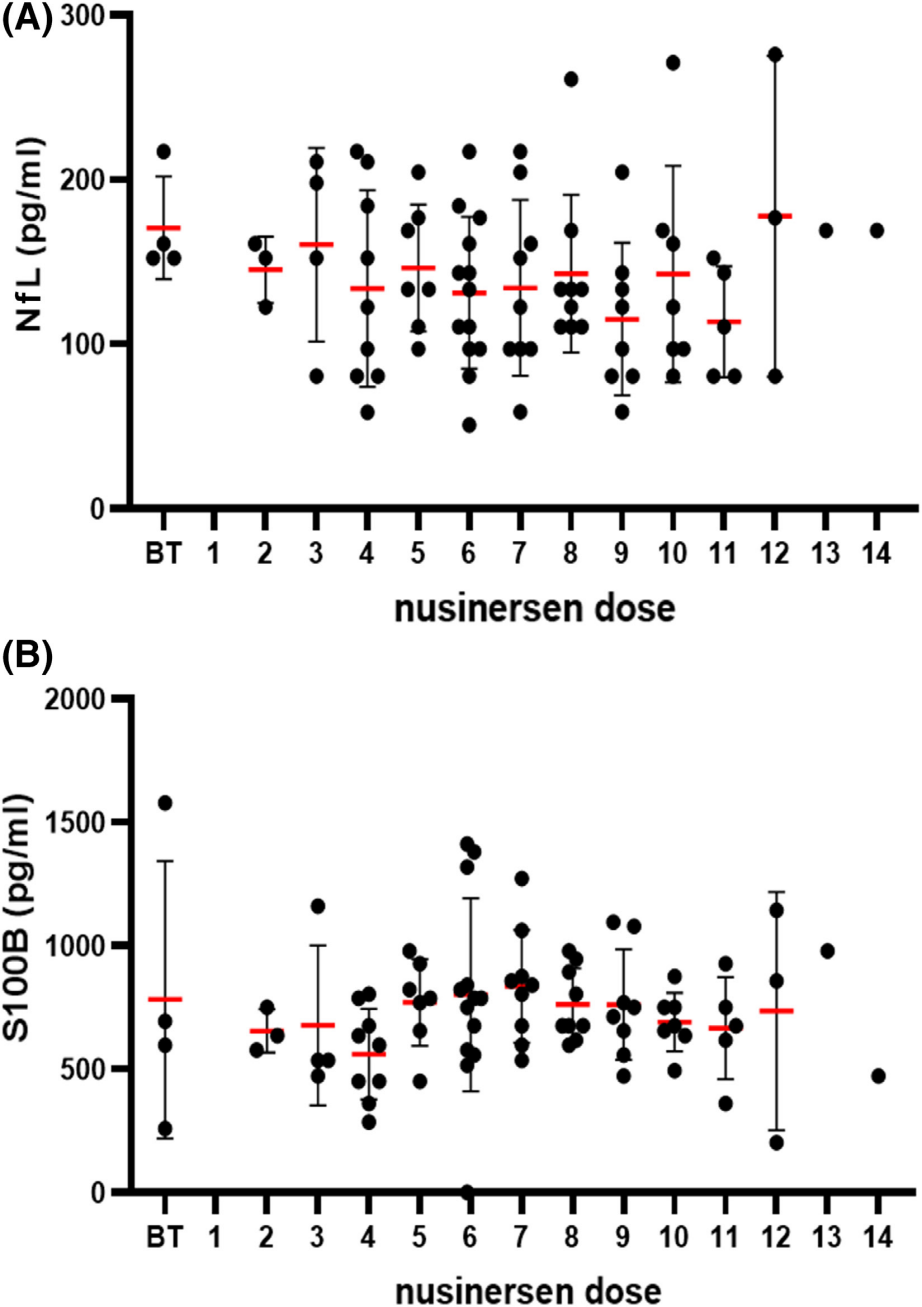
(A) NfL and (B) S100B levels in all SMA patients (with SMA 1, SMA 2, and SMA 3) who received different doses of nusinersen. Each patient is represented by a dot on the graph. The red horizontal line represents the mean value, while the black horizontal lines represent the standard deviation. *p < 0.05

The NfL protein values were overall not significantly decreased between baseline values and after a different number of nusinersen doses (*H* test = 9.67; *df* = 13; *p* = 0.72; Figure 3) in all SMA patients. The same result was confirmed by linear regression (β = −0.08; SE = 1.78; *p* = 0.49). In patients with SMA type 1 who received a different number of nusinersen doses, the NfL protein levels did not differ significantly (*H* test = 11.41; *df* = 13; *p* = 0.58). Likewise, in patients with SMA types 2 and 3 who received a different number of nusinersen doses, the NfL protein values did not differ significantly (*H* = 6.95; *df* = 11; *p* = 0.80).

In patients with SMA of all three types who received a different number of nusinersen doses, the S100B protein levels did not differ significantly from baseline values (*H* test = 13.33; *df* = 13; *p* = 0.42; Figure 3) in all SMA patients. This was confirmed by linear regression (β = 0.03; SE = 9.54; *p* = 0.42). In patients with SMA type 1 who received a different number of nusinersen doses, S100B protein levels did not differ significantly (*H* test = 14.91; *df* = 13; *p* = 0.31).

### 3.4 CHOP INTEND and HFMSE scores in patients with SMA of all three types

**FIGURE 4:**
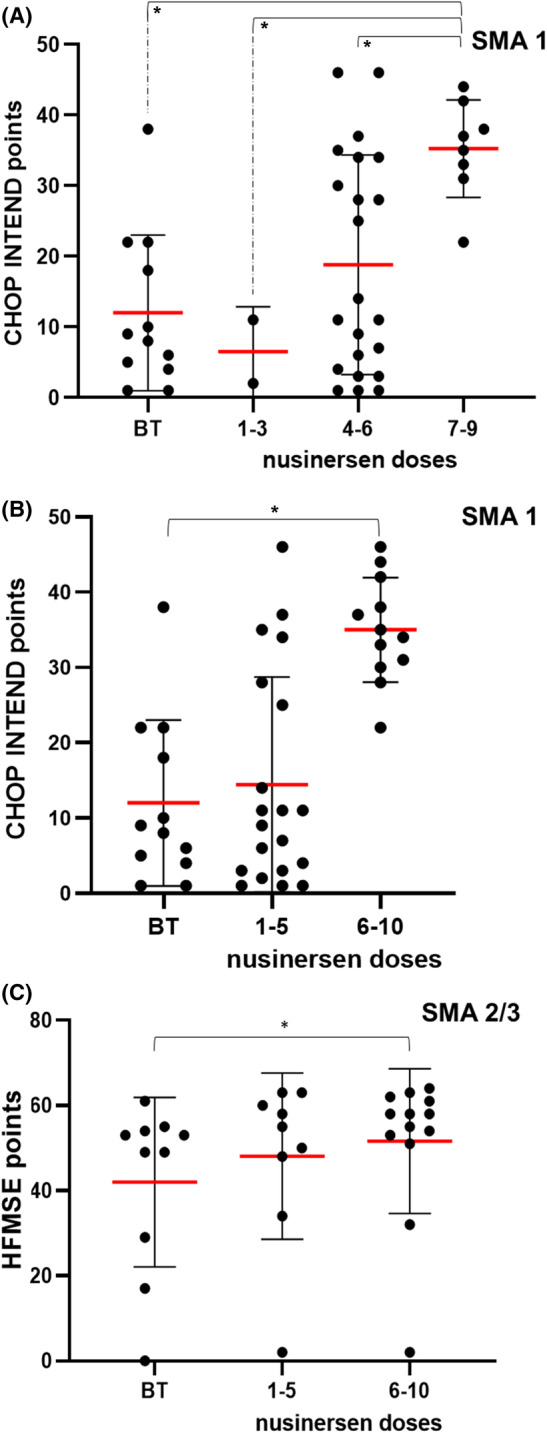
(A and B) CHOP INTEND scores and (C) HFMSE scores and correlation with the number of nusinersen doses. Each patient is represented by a dot on the graph. The red horizontal line represents the mean value, while the black horizontal lines represent the standard deviation. *p < 0.05

A statistically significant difference in the number of points on the CHOP INTEND scale was seen between SMA type 1 patients who received a different number of nusinersen doses (*H* test = 17.9; *df* = 8; *p* = 0.02; Figure 4). The most significant differences were seen between SMA type 1 patients who received the 6th, 7th, 8th, and 9th dose of nusinersen compared to baseline values (Table 4).

**TABLE 4:** Statistical comparison of CHOP INTEND scores in analyzed groups

Tested groups	The result of the statistical test
SMA 1	
BT vs. 6th dose	*U* = 3; *Z* = −2.61; *p* = 0.01
BT vs. 7th dose	*U* = 8; *Z* = −2.05; *p* = 0.04
BT vs. 8th dose	*U* = 1.50; *Z* = −2.43; *p* = 0.01
BT vs. 9th dose	*U* = 1; *Z* = −2.04; *p* = 0.03
BT vs. 7th–9th dose	*U* = 6.5; *Z* = −3.21; *p* < 0.01
1.–3rd dose vs. 7th–9th dose	*U* = 0; *Z* = −2.12; *p* = 0.04
4.–6th dose vs. 7th–9th dose	*U* = 50.50; *Z* = −2.33; *p* = 0.02
BT vs. 6.–10. dose	*U* = 9.5; *Z* = −3.61; *p* < 0.01
1.–5th dose vs. 6.–10. dose	*U* = 49; *Z* = −3.21; *p* < 0.01

Abbreviations: BT, before therapy; SMA, spinal muscular atrophy.

**FIGURE 5:**
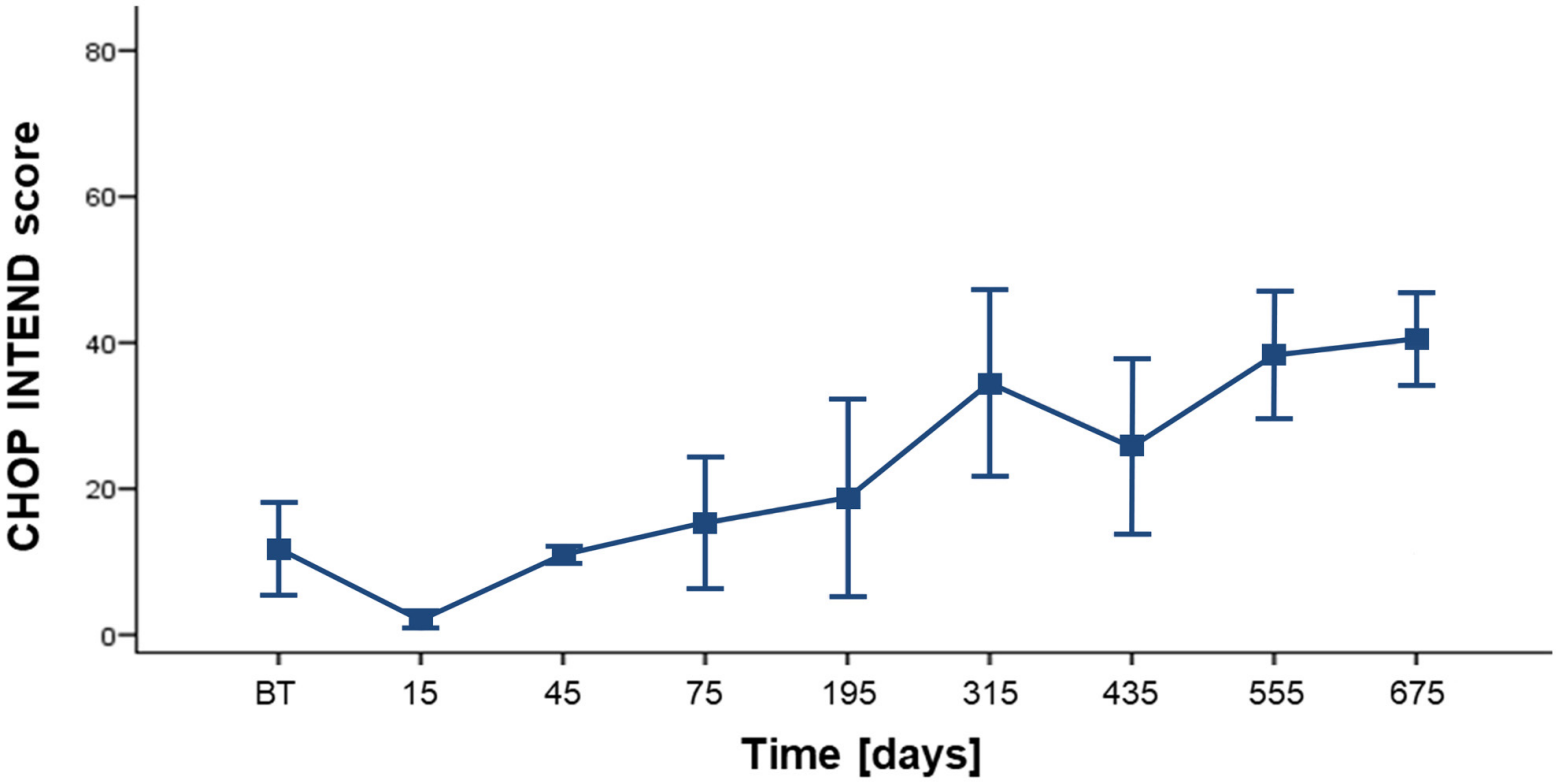
CHOP INTEND scores relative to the baseline scores

Patient data were also analyzed in four groups (before treatment, 1–3 doses group, 4–6 doses group, and 7–9 doses group). CHOP INTEND values differed significantly in patients who received different numbers of nusinersen doses (*H* test = 11.74; *df* = 3; *p* = 0.01; Figure 5). The CHOP INTEND value was significantly higher in patients in the 7th to 9th dose group (second year of therapy or 12–24 months after therapy onset) compared to baseline values, in patients in the group from 1st to 3rd dose, and in the group from 4th to 6th dose (Figure 4 and Table 4).

A statistically significant difference was also observed in HFMSE scores between patients with SMA types 2 and 3 who were administered a different number of nusinersen doses (Figure 4). The reason for this was the fact that nusinersen could not be administered intrathecally in some patients due to scoliosis. Compared to the scores attained before the treatment, the most significant differences were seen in patients who received the 8th dose (*U* = 1; *Z* = −2.37; *p* = 0.02), the 6th to 10th dose (*U* = 32.5; *Z* = −2.02; *p* = 0.04), and after 12 months of total treatment duration (*U* = 1; *Z* = −2.37; *p* = 0.02).

## 4 DISCUSSION

This longitudinal study aimed to determine the effect of nusinersen on three selected biological markers in CSF of SMA patients. The duration of the follow‐up in our study is longer than the previous studies (75–1275 days, on average 675 days). This longitudinal design allowed us to study changes in CSF biological markers and the CHOP INTEND and HFMSE scores at multiple time points, with up to 15 nusinersen doses. The studies with the longest follow‐up period were conducted by Nitz et al. (34 months or 1034 days)^41^ and Olsson et al. (patients received eight doses over approximately 435 days).^38^ All other studies followed SMA patients for up to 195 days (or six nusinersen doses). An additional advantage of our study is the fact that we included as many as 30 genotyped patients with different types of SMA (types 1, 2, and 3). We showed that treatment with nusinersen resulted in significant motor improvement as indicated by the increase in CHOP INTEND and HFMSE scores. Among the analyzed CSF biomarkers, only t‐tau protein showed to be a reliable biological marker for monitoring the response to nusinersen therapy, especially in the first 18–24 months of therapy when it significantly decreased from baseline levels in all three SMA types, although in the longer term, this relationship was no longer maintained. The NfL protein showed no significant reduction in CSF independent of the number of nusinersen doses, and the S100B protein CSF levels did not correlate with either the nusinersen doses or the assessed motor function measurement scores.

To date, only five studies analyzed CSF t‐tau concentrations after nusinersen treatment in SMA,^25^, ^38^, ^39^, ^43^, ^44^ but none of these studies followed SMA patients as long as we did. Olsson et al. and Johannsen et al. reported results similar to ours and detected the most significant decrease in t‐tau concentrations in SMA type 1 and 2 patients following treatment, which also correlated well with improvements in motor abilities.^38^, ^39^ Other studies did not detect significant changes in t‐tau concentrations as a result of treatment.^25^, ^43^, ^44^ These studies mainly included SMA type 2 and 3 patients, while our study and those of Olsson et al.^38^ and Johannsen et al.^39^ included patients with SMA type 1 as well. Johanssen et al. observed a significant decrease in t‐tau levels in patients with SMA type 1 with an average age of 16 months and SMA type 2 with an average age of 78.8 months.^39^ Olsson et al. observed a significant decrease in t‐tau levels after administration of nusinersen in SMA type 1 patients with an average age of 2.2 months.^38^ Studies that did not observe a change in t‐tau levels mainly included SMA type 2 and type 3 patients who started nusinersen treatment at an older age: SMA type 2 and type 3, the median age at the beginning of the therapy was 41 years,^44^ SMA 3, the median age at the beginning of the therapy was 39 years,^25^ and SMA 3, the median age at the beginning of the therapy was 12 years, respectively.^43^ Moreover, in our study, we did not observe a change in t‐tau levels upon nusinersen treatment in patients with SMA type 2 and type 3. Therefore, it is likely that an earlier start of the treatment with nusinersen results in a significant decrease in CSF t‐tau. While the role of tau protein changes in mechanisms underlying motoneuron degeneration in SMA remains largely unknown^54^ based on results presented in this and other previously mentioned reports,^38^, ^39^ it appears that the measurement of t‐tau in CSF is a valuable tool for monitoring the response to nusinersen in SMA patients.

Several studies have analyzed the influence of nusinersen on NfL protein concentrations in CSF.^38^, ^39^, ^40^, ^41^, ^44^ In agreement with the present findings, Milella et al. and Rich et al. found no effect,^44^, ^55^ whereas others reported significantly reduced values of NfL protein in CSF after the administration of nusinersen.^38^, ^39^, ^40^, ^41^, ^42^ These outcomes reflect the large variance among patients regarding this potential marker, making it a weak candidate for monitoring response to nusinersen treatment.

The concentration of S100B (together with NfH and NSE) was analyzed in a single study in CSF of 11 patients with SMA type 3 after the administration of nusinersen.^25^ No changes in the S100B value in the patients' CSF were detected after the administration of nusinersen in that study as well.

A shortcoming of this study is the lack of a control group of patients/respondents. However, lumbar puncture is considered an invasive procedure and is performed on healthy subjects only in exceptional circumstances. Despite this limitation, the conclusions derived from this study retain significance in monitoring nusinersen treatment in patients with SMA.

## 5 CONCLUSION

Our study confirms that nusinersen therapy is associated with an increase in the functional motor scales' scores. This result is consistent with the other studies that reported a comparable outcome after the administration of nusinersen.^25^, ^38^, ^39^, ^41^, ^43^, ^56^, ^57^, ^58^, ^59^, ^60^

Even though NfL reduction in CSF had been reported in a few studies of SMA patients on nusinersen, NfL, in general, does not seem to represent a reliable biomarker to monitor the response of SMA patients to nusinersen treatment. Finally, our study shows that t‐tau concentration is emerging as a reliable biomarker for monitoring the response of SMA patients to nusinersen treatment in the first 2‐year period.

## AUTHOR CONTRIBUTIONS

Design and conceptualization: GŠ and NB; Data collection: VV, ATG, MBK, AMŠ, ŽV, IL, JS, NB, and GŠ; Methodology: VV, MBab, MBan, IB, EŠ, KZ, ATG, AMŠ, JS, NB, and GŠ; Investigation and formal data analysis: VV, MBab, MBan, IB, PRH, NB, and GŠ; Visualization: MBab, MBan, IB, and GŠ; Resources: NB and GŠ; Writing – original draft preparation: VV, MBab, MBan, IB, PRH, JS, NB, and GŠ; Writing – review and editing: all coauthors; Supervision: NB and GŠ.

## FUNDING INFORMATION

This work was funded by The University of Zagreb grant BM99/2020 to GŠ (“Blood and cerebrospinal fluid biomarkers in brain diseases”), the Croatian Science Foundation grant IP‐2019‐04‐3584 to GŠ (“Role of the blood‐brain barrier, innate immunity, and tau protein oligomerization in the pathogenesis of Alzheimer's disease”), and the Scientific Centre of Excellence for Basic, Clinical, and Translational Neuroscience CoRE‐NEURO (“Experimental and clinical research of hypoxic‐ischemic damage in perinatal and adult brain”; GA KK01.1.1.01.0007 funded by the European Union through the European Regional Development Fund).

## CONFLICT OF INTEREST

Dr. Goran Šimić is an Editorial Board member of CNS Neuroscience and Therapeutics and a coauthor of this article. To minimize bias, they were excluded from all editorial decision‐making related to the acceptance of this article for publication.

## Data Availability

All relevant data are presented in this article. Original data are available from the corresponding author upon reasonable request.
